# On the Role of Self-compassion and Self-kindness in Weight Regulation and Health Behavior Change

**DOI:** 10.3389/fpsyg.2017.00229

**Published:** 2017-02-16

**Authors:** Michail Mantzios, Helen H. Egan

**Affiliations:** Department of Psychology, Birmingham City UniversityBirmingham, UK

**Keywords:** weight regulation, self-care, self-compassion, self-kindness, mindfulness, eating behaviors

The construct of self-compassion has been investigated in relation to health behaviors, health behavior change, and health outcomes such as regulated eating and weight loss. Self-compassion has been defined as a mindful awareness of oneself, which involves treating oneself kindly and understanding oneself during difficult and challenging times by realizing that such experiences are common amongst all humans (Neff, 2003a). Neff (2003a,b) described how self-compassion consists of three interrelated components: *self-kindness (vs. self-judgment), common humanity (vs. isolation)*, and *mindfulness (vs. over-identification)*. While the psychological benefits are well documented (e.g., Neff, 2011), the health behaviors and outcomes may require more consideration, and this opinion manuscript aims to shed light on potential problems in eating and weight issues. Initial findings of self-compassion in assisting regulated eating are promising, and are explored next.

## Self-compassion and eating

Empirical studies investigating self-compassionate-based interventions have generally focused on weight management. For example, a recent study found that the trait of self-compassion negatively predicted weight gain in civilians who entered a highly stressful military environment (Mantzios et al., 2015). Another study found that participants lost more weight when they participated in a mindful self-compassionate programme, compared to a control group (Mantzios and Wilson, 2015). Participants in these studies were not typical populations that are usually investigated in weight loss and weight regulation trials. They exercised regularly and had a controlled diet, while the confined environment created a very particular experimental setting which resulted in restricting generalizability of these findings; hence, further research is needed that can be more readily extrapolated. In an attempt to induce self-compassion in a mindfulness context that was more behavior-specific than mindfulness meditation, Mantzios and Wilson (2014) developed a “mindful concrete construal” diary. The diary was an event-based exercise to be used with every meal, and aimed in increasing mindfulness and self-compassionate levels when eating. The diary emphasized mindfulness and self-compassion, and created a tool for participants to observe goals that were visible and achievable in the present moment, while being kind, accepting, and understanding to thoughts and feelings that arose during each meal. The diary was successful in producing similar levels of weight loss to a meditation group, and improved the delay in weight regain. Initial findings were significant and promising, but follow-up research is warranted.

Explanations vary as to how self-compassion may support eating behaviors, behavior change, and weight regulation. Mantzios and Wilson (2014) proposed that self-compassion may amplify the effectiveness of mindfulness, while other research suggested that self-compassion relates to mindful eating (Webb et al., 2013), intuitive eating (Schoenefeld and Webb, 2013), overcoming disinhibition when dieting (Adams and Leary, 2007), and increasing health behavior intentions (Sirois, 2015). Again, despite the positive findings, self-compassion still remains a trait that requires further research, merely because it is uncertain whether self-compassion leads to healthy choices around food, and which components are more helpful or may need emphasis when developing interventions.

## Differentiating between self-compassion and self-kindness

Unfortunately the questions that exist in the field are not the only concerns that we identified in contemporary research that involves the role of self-compassion in health and regulated eating. First, many people—including researchers and clinicians—think that self-compassion is the same as self-kindness, and that all components measured with the self-compassion scale are equally positive toward health behavior change. This assumption then suggests that being kinder to yourself supports weight regulation and eating behaviors. Currently popular media and self-help books enthusiastically propose that being kinder to yourself is beneficial to people who are attempting to regulate their weight, but the benefits for the physiological health is questionable. In other words, synonymising and conforming kindness with compassion leads to pitfalls in contemporary health research, and is not consistent with Neff's conceptualization of self-compassion.

Recruiting participants within the UK and asking them how they show kindness to themselves clearly showed that there are many individual differences in behavioral enactments of self-kindness (Mantzios and Egan, submitted). Our recent findings show that self-kindness can take many forms, and some people described acts of self-kindness to be watching television, indulge and over-indulge on their favorite foods, use recreational drugs, and binge drink. Others said that to be kind to themselves they might take a warm bath, go for a walk, jog, or eat a healthy and nourishing meal. The former group of behaviors may have negative health consequences (e.g., lead to obesity, cardiovascular disease), while the latter group of behaviors depicts an alignment of self-kindness and self-care, which is perhaps a truer model of self-compassion, and has been briefly mentioned in past literature (Neff, 2009, 2011), and relates to the body and mind simultaneously.

Second, and reflective of our recent findings, methodological and measurement issues present another reason why self-kindness should be recommended with caution to people who seek support with health behaviors. Items in the self-compassion scale such as “I'm kind to myself when I'm experiencing suffering” and “When I'm going through a very hard time, I give myself the caring and tenderness I need” are open to interpretation, and do not tell us anything about how that kindness is enacted in everyday life. In fact, health behavior items that reflect self-kindness, or a measure that accounts for both psychological and physiological wellbeing, either separate or combined with the self-compassion scale may assist in gaining a holistic version of self-kindness, and thus, of self-compassion, which may be more predictive of health behaviors and health behavior change. Again, self-kindness should not be perceived as being self-compassion.

## Association of self-compassion with other health theories and research

Other research has shown how people use a justification-based model, whereby they legitimize the surpassing of long-term goals and rationally gratify immediate needs (see De Witt Huberts et al., 2014). A justification has been recognized as a method of increasing (a) the choice of unhealthy (over healthy) products, and (b) the consumption of food (De Witt Huberts et al., 2012a,b). For example, in a recent study, participants were shown aversive pictures as a method of inducing a negative affective state. Participants who were highly induced to experience negative affect consumed more snack foods at the subsequent taste test (De Witt Huberts et al., 2012a). In this study, it could be assumed that participants were at a lower spectrum of the self-compassionate threshold, but that does not necessarily entail that they were not being kind to themselves. Whether participants were being kind to themselves (or not) remains a question that needs further consideration; however, the kindness shown toward the emotional/affective continuum might be apparent. Whether these findings relate to justification or not, mindfulness or avoidance, or, genuine or false interpretations of self-compassion, the truth is that an emotional eater (i.e., eats in response to his/her emotions), is mainly using this method to be kind to him or herself (at least partially since there is no consideration of long-term health consequences and consecutive feelings of shame and/or guilt). In other words, the behavioral enactment of self-kindness may often relate to food consumption.

In further support, interpersonal conflict and/or goal conflict theories (Strack and Deutsch, 2004; Tangney et al., 2004; Carver, 2005; Stroebe et al., 2008) may well relate to a “novel” self-compassionate model for health. For example, the reflective system, which employs high-order executive functioning to control decisions and actions, may be responsible for “treating” or caring for the mind over the body. Similarly, the wellbeing of the body and mind may at times be two incompatible goals. Contrary to a hedonic (low-order) vs. control paradigm (high-order) paradigm, the equal status of both body and mind are operationalized in terms of immediacy over long-term goals and reasoned actions. In other words, time and again, in a very conscious mind frame, we give our child the chocolate, because the mind is under distress, and the care of the body can be resumed some time later. Therefore, research from various health disciplines should not be ignored or disregarded, as such research may dictate future research and new avenues for self-compassionate research in eating and health psychology.

## Future directions

The root of the problem appears to be a wide separation between psychological and physiological health within the notion of self-kindness, and in effect, of self-compassion. This is not to say that self-kindness is unhelpful in health, but that it may moderate positive relationships. Self-kindness has been considered thus far a positive method of alleviating psychological distress, by investigating the affective/cognitive elements of self-kindness. However, being kind to yourself does not necessarily mean that both psychological and physiological health are achieved. In practice, we note that people frequently prioritize psychological over physiological health and vice versa. Consider a scenario where you are on a weight loss diet, you get home after a very stressful and demanding day at work, and in a fatigued and depleted state you seek some relaxation, comfort and peace. You want to treat yourself to your favorite chocolate bar. The stress and frustration experienced justifies the urgency or priority over the long-term goal of weight loss. At the end of the day, “you deserve it” (Taylor et al., 2014) and you should have it “because you are worth it” (De Witt Huberts et al., 2014). Psychological health is then cared for over the physiological health due to the immediacy of the situation. While we could argue that in this scenario the protagonist is not self-compassionate or mindful, we could argue that he/she is displaying kindness to himself/herself. Whether this act of self-kindness relates to food, a glass of wine, or, smoking a cigarette, there is often a detrimental health aspect and this warrants further investigation.

Overall, it seems that some behaviors (such as eating high calorie and low nutritional foods), comfort and sooth the mind, but may be, especially in excess, uncaring and damaging to the body and physiological health of the individual. This appraisal of the literature is by no means a critique of the self-compassion scale, but rather a realization that physiological health may be “put on the back burner” when we care for our mind. In considering theories of self-compassion and self-kindness—and even further theories of acceptance and mindfulness —there is a need to emphasize holistic self-care, as it appears that these are key components in alleviating the suffering for both the body and mind (Kabat-Zinn, 2003). Incorporating holistic self-care (i.e., caring equally for the body and mind, or, psychological and physiological health) in contemporary investigations of self-kindness in health contexts, and thus, of self-compassion, is necessary and important in facilitating behavioral change.

## Ethics statement

This article does not contain any studies with human participants performed by any of the authors.

## Author contributions

MM wrote the initial draft and conceptualized the fundamentals of this manuscript. HE reviewed the manuscript several times and contributed to discussions and clarifications around the topics discussed; this way, greatly contributing toward the final formation of ideas and concepts presented in the manuscript.

### Conflict of interest statement

The authors declare that the research was conducted in the absence of any commercial or financial relationships that could be construed as a potential conflict of interest.

## References

[B1] Adams C. E. Leary M. R. (2007). Promoting self-compassionate attitudes toward eating among restrictive and guilty eaters. J. Soc. Clin. Psychol. 26, 1120–1144. 10.1521/jscp.2007.26.10.1120

[B2] Carver C. S. (2005). Impulse and constraint: perspectives from personality psychology, convergence with theory in other areas, and potential for integration. Pers. Soc. Psychol. Rev. 9, 312–333. 10.1207/s15327957pspr0904_2 16223354

[B3] De Witt Huberts J. C. Evers C. De Ridder D. T. (2012a). License to sin: self-licensing as a mechanism underlying hedonic consumption. Eur. J. Soc. Psychol. 42, 490–496. 10.1002/ejsp.861

[B4] De Witt Huberts J. C. Evers C. De Ridder D. T. D. (2012b). I Did Good So Now I Can be Bad. Justifications Elicit a Hedonic Orientation in Restrained Eaters. Published Thesis. Available online at: http://dspace.library.uu.nl/bitstream/handle/1874/258625/witt-huberts.pdf?sequence=2#page=95

[B5] De Witt Huberts J. C. Evers C. De Ridder D. T. (2014). “Because I Am Worth It” a theoretical framework and empirical review of a justification-based account of self-regulation failure. Pers. Soc. Psychol. Rev. 18, 119–138. 10.1177/1088868313507533 24214148

[B6] Kabat-Zinn J. (2003). Mindfulness-based interventions in context: past, present, and future. Clin. Psychol. 10, 144–156. 10.1093/clipsy.bpg016

[B7] Mantzios M. Wilson J. C. (2014). Making concrete construals mindful: a novel approach for developing mindfulness and self-compassion to assist weight loss. Psychol. Health 29, 422–441. 10.1080/08870446.2013.863883 24215123

[B8] Mantzios M. Wilson J. C. (2015). Exploring mindfulness and mindfulness with self-compassion-centered interventions to assist weight loss: theoretical considerations and preliminary results of a randomized pilot study. Mindfulness 6, 824–835. 10.1007/s12671-014-0325-z

[B9] Mantzios M. Wilson J. C. Linnell M. Morris P. (2015). The role of negative cognition, intolerance of uncertainty, mindfulness, and self-compassion in weight regulation among male army recruits. Mindfulness 6, 545–552. 10.1007/s12671-014-0286-2

[B10] Neff K. D. (2003a). Self-compassion: an alternative conceptualization of a healthy attitude toward oneself. Self Identity 2, 85–101. 10.1080/15298860309032

[B11] Neff K. D. (2003b). The development and validation of a scale to measure self-compassion. Self Identity 2, 223–250. 10.1080/15298860309027

[B12] Neff K. D. (2009). The role of self-compassion in development: a healthier way to relate to oneself. Hum. Dev. 52, 211–214. 10.1159/000215071 22479080 PMC2790748

[B13] Neff K. D. (2011). Self-compassion, self-esteem, and well-being. Soc. Pers. Psychol. Compass 5, 1–12. 10.1111/j.1751-9004.2010.00330.x

[B14] Schoenefeld S. J. Webb J. B. (2013). Self-compassion and intuitive eating in college women: examining the contributions of distress tolerance and body image acceptance and action. Eat. Behav. 14, 493–496. 10.1016/j.eatbeh.2013.09.001 24183143

[B15] Sirois F. M. (2015). A self-regulation resource model of self-compassion and health behavior intentions in emerging adults. Prevent. Med. Rep. 2, 218–222. 10.1016/j.pmedr.2015.03.006 26844074 PMC4721380

[B16] Strack F. Deutsch R. (2004). Reflective and impulsive determinants of social behavior. Pers. Soc. Psychol. Rev. 8, 220–247. 10.1207/s15327957pspr0803_1 15454347

[B17] Stroebe W. Mensink W. Aarts H. Schut H. Kruglanski A. W. (2008). Why dieters fail: testing the goal conflict model of eating. J. Exp. Soc. Psychol. 44, 26–36. 10.1016/j.jesp.2007.01.005

[B18] Tangney J. P. Baumeister R. F. Boone A. L. (2004). High self-control predicts good adjustment, less pathology, better grades, and interpersonal success. J. Pers. 72, 271–324. 10.1111/j.0022-3506.2004.00263.x 15016066

[B19] Taylor C. Webb T. L. Sheeran P. (2014). “I deserve a treat!”: justifications for indulgence undermine the translation of intentions into action. Br. J. Soc. Psychol. 53, 501–520. 10.1111/bjso.12043 23855962

[B20] Webb J. B. Jafari N. Schoenefeld S. J. Hardin A. S. (2013). Examining a model of self-compassion, body shame, and mindful eating, in Poster Presented at the Society of Behavioral Medicine's 34th Annual Meeting and Scientific Sessions (San Francisco, CA).

